# *CAPN1* Variants as Cause of Hereditary Spastic Paraplegia Type 76

**DOI:** 10.1155/2019/7615605

**Published:** 2019-07-01

**Authors:** Jesus Eduardo Garcia-Berlanga, Mariana Moscovich, Isaac Jair Palacios, Alejandro Banegas-Lagos, Augusto Rojas-Martinez, Daniel Martinez-Ramirez

**Affiliations:** ^1^Tecnologico de Monterrey, Escuela de Medicina y Ciencias de la Salud, Monterrey, Nuevo Leon, Mexico; ^2^Department of Neurology, Christian-Albrechts University, Kiel, Germany

## Abstract

**Background:**

Autosomal recessive hereditary spastic paraplegias (HSP) are a rare group of hereditary neurodegenerative disorders characterized by spasticity with or without other symptoms.* SPG11 *gene is the most common cause of autosomal recessive HSP. We report a case of autosomal recessive spastic paraplegia type 76 due to heterozygous variants of* CAPN1* in an Argentinean subject.

**Case Presentation:**

A 38-year-old Argentinean female presented with progressive gait problems and instability of 15-year duration. Oculomotor abnormalities, ataxia, bradykinesia, cervical dystonia, and lower limb pyramidal signs were observed. Brain MRI was unremarkable. Whole-exome sequencing analysis identified two heterozygous variants in* CAPN1*.

**Conclusions:**

Clinicians should screen for* CAPN1* mutation in a young female patient without significant family history with a spastic paraplegia syndrome associated with other symptoms.

## 1. Background

Hereditary spastic paraplegias (HSP) are a group of heterogeneous degenerative disorders characterized by lower limb spasticity and weakness due to progressive degeneration of corticospinal tracts [1]. HSP can present as a pure form only with pyramidal symptoms, or as a complex form associated with other symptoms. HSP are transmitted in all modes of inheritance [2]. The autosomal dominant mode of inheritance is the most prevalent representing 70% of cases. Mutation in* SPAST* gene accounts for 40% of the autosomal dominant HSP. In the recessive HSP, the most frequent mutation is in* SPG11*. We report a case of autosomal recessive spastic paraplegia type 76 (SPG76, OMIM #616907) due to heterozygous variants of* CAPN1* in an Argentinean subject.

## 2. Case Presentation

A 38-year-old Argentinean female presented with slowly progressive unsteadiness noticed first at age 23. She reported pronounced instability and gait problems as disease progressed. Her gait problems were described as short steps, with starting hesitation, fear of falling, and needing to hold from walls to avoid falling. She also reported several falls, dizziness, neck pain, and constipation. Symptoms progressed over the years affecting her mobility and functionality. She currently needs assistance for moving around. No relevant medical, family, or psychosocial history was reported. No past interventions were reported.

On neurological examination (Video 1), she presented dysarthria, interrupted slow horizontal and vertical eye movements, and slow horizontal saccades. She manifested spasticity and hyperreflexia more pronounced in her lower extremities. Mild cervical dystonia with bradykinesia was also observed. She showed ataxic symptoms more pronounced on her left upper extremity. Gait was spastic and no cognitive abnormalities were observed.

Brain MRI with and without contrast was unremarkable. Due to the presence of a slowly progressive adult onset spastic-ataxia syndrome, associated with other neurological abnormalities, and facing the challenge of poor financial access, we decided to optimize our resources studying the patient using whole-exome sequencing (CentoDX™, Centogene AG, Germany). The analysis identified two variants in* CAPN1* (MIM:114220) considered as probably pathogenic Class 2, according to the American College of Medical Genetics and Genomics criteria. She was heterozygous for a splicing mutation in intron 16 (c.1729+1G>A) and a second splicing mutation in intron 12 (c.1353+2T>C). Carrier testing in the parents was not performed. Due to the strong phenotypic overlap between the symptoms and previously reported cases, we consider the detected variants as pathogenic of SPG76.

## 3. Discussion

We report two pathogenic variants of* CAPN1* gene and the first case affecting two noncoding regions (introns) in a Latin-American patient.  Table 1 describes all SPG76 reported cases in the literature [3, 4]. We observed that female patients are more commonly (67%) affected, with a mean age of onset of 19.8 years (Min. = 5, Max. = 39), most had family history of consanguinity (71%), and most were homozygous (77%). All initiated with lower limb spasticity, 85% reported upper limb spasticity, 58% showed ataxia, and 41% reported dysarthria. Our case also presented with oculomotor abnormalities. Three cases showed cerebellar atrophy and 1 spinal atrophy on MRI.

In comparison with other published cases, we found similarities in that all of them presented lower limb spasticity and ataxia. The difference from our case was the oculomotor abnormalities, which was also reported in only one other case [5]. We suggest that the combined phenotype of spasticity and ataxia with oculomotor abnormalities, in a young female patient of Arab origin, could be a diagnostic clue for SPG76. The age of onset of our case was similar to that previously reported. All of the subjects experienced pronounced instability and gait problems as disease progresses [6].

*CAPN1* mutations account for 2.2% of autosomal recessive HSP.* CAPN1* is located in chromosome 11q13 and encodes calpain 1, a calcium-activated cysteine protease that is widely present in the central nervous system. The exact role of calpain 1 in humans is unclear; however, studies in animal models suggest that calpain 1 is involved in synaptic plasticity, neuronal migration, neuronal necrosis, and maintenance [7].

## 4. Conclusions

Our report adds to the clinical and genetical spectrum of* CAPN1*-related SPG76 disorders. We recommend clinicians to consider screening for* CAPN1* in a young female patient with spastic paraplegia with additional neurological symptoms without significant family history.

## Figures and Tables

**Table 1:** Clinical and genetical characteristics of SPG76 cases reported in the literature.

	Ethnicity	Age at onset	Age at diagnosis	Gender	Lower limbs spasticity	Upper limbs spasticity	Ataxia	Dysarthria	Oculomotor Impairment	Exon or Intron affected	Mutation	Type	Heterozygous /Homozygous	Consanguinity	Brain MRI	NCS and SSEP
1	Latin American	NA	NA	NA	+	+	-	-	-	-	c.1176G>A p.Trp392∗	Stop gain mutation	Homozygous	+	NA	NA

2	Latin American	NA	NA	NA	+	+	-	-	-	-	c.1176G>A p.Trp392∗	NA	Homozygous	+	NA	NA

3	Latin American	NA	NA	NA	+	+	-	-	-	-	c.1176G>A p.Trp392∗	NA	Homozygous	+	NA	NA

4	Latin American	22	37	F	+	+	-	-	-	-	c.1176G>A p.Trp392∗	NA	Homozygous	-	NA	NA

5	Caucasian	20	46	F	+	+	+	-	-	-	c.675C>A p.Tyr225∗	Novel LoF Mutation	Homozygous	-	NA	NA

6	Caucasian	35	51	M	+	+	+	-	-	-	c.675C>A p.Tyr225∗	Novel LoF Mutation	Homozygous	-	NA	NA

7	Latin American	30	42	F	+	+	+	-	-	-	c.1176G>A p.Trp392∗ c.618_619delAG p.Gly208Glnfs∗7	LoF Mutation	Heterozygous	+	NA	NA

8	Latin American	38	-	M	+	+	+	-	-	-	c.1176G>A p.Trp392∗	LoF Mutation	Homozygous	+	NA	NA

9	Arab	20	31	F	+	+	-	+	-	Ex:8	c.884G>C p.Arg295Pro	LoF Mutation	Homozygous	+	NA	Normal

10	Arab	NA	NA	NA	NA	NA	NA	NA	NA	Ex:8	c.884G>C p.Arg295Pro	LoF Mutation	Homozygous	+	NA	NA

11	Arab	NA	NA	NA	NA	NA	NA	NA	NA	Ex:8	c.884G>C p.Arg295Pro	LoF Mutation	Homozygous	+	NA	NA

12	Arab	35	47	M	+	+	-	+	-	Ex:14	c.1579C>T p.Gln527	Stop variant	Homozygous	+	NA	Moderate Sensory Axonal Neuropathy

13	Arab	36	44	F	+	+	+	+	-	Ex:14	c.1579C>T p.Gln527	Stop variant	Homozygous	+	NA	Moderate Sensory Axonal Neuropathy

14	Arab	22	42	M	+	+	-	+	-	Ex:14	c.1579C>T p.Gln527	Stop variant	Homozygous	+	Normal	NA

15	Arab	39	40	M	+	+	+	+	-	Ex:14	c.1579C>T p.Gln527	Stop variant	Homozygous	+	NA	NA

16	Arab	NA	NA	NA	NA	NA	NA	NA	NA	Ex:14	c.1579C>T p.Gln527	Stop variant	Homozygous	+	NA	NA

17	Arab	NA	NA	NA	NA	NA	NA	NA	NA	Ex:14	c.1579C>T p.Gln527	Stop variant	Homozygous	+	NA	NA

18	Arab	NA	NA	NA	NA	NA	NA	NA	NA	Ex:14	c.1579C>T p.Gln527	Stop variant	Homozygous	+	NA	NA

19	Caucasian	24	30	F	+	-	-	-	-	Ex:4	c.406delC p.Pro136Argfs∗40	Deletion	Heterozygous	-	NA	Normal

20	Caucasian	33	35	M	+	-	-	-	-	Ex:4	c.406delC p.Pro136Argfs∗40	Deletion	Heterozygous	-	Atrophy of spinal cord	NA

21	Caucasian	19	22	F	+	+	+	-	-	Ex:4	c.1605+5G>A	Mutation Splicing	Heterozygous	-	Normal	NA

22	Indian	33	43	F	+	+	+	+	-	Ex:3	c.337+1G>A	Splice Mutation	Homozygous	+	Mild cerebellar atrophy	Normal

23	Indian	NA	NA	F	NA	NA	NA	NA	NA	Ex:3	c.337+1G>A	Splice Mutation	Homozygous	+	NA	NA

24	Caucasian	29	39	F	+	+	+	+	+	Ex:6	c.759+1G>A	Donor splice site	Homozygous	+	Mild cerebellar vermal atrophy	NA

25	Caucasian	33	37	F	+	+	+	+	-	Ex:6	c.759+1G>A	Donor splice site	Homozygous	+	NA	NA

26	Caucasian	5	16	M	+	-	-	-	-	-	c.221GNA/ p.(G74D) c.911CNT/ p.(T304M) c.1418GNT/ p.(R473L)	missense	Heterozygous	-	Normal	Normal

27	Arab	21	37	F	+	+	+	+	-	-	c.994G>A P.Gly.332Arg	NA	Homozygous	+	Normal	Normal

28	Arab	30	54	F	+	+	+	+	-	Ex10	c. 1176G>A p. Trp392	Nucleotide substitution	Heterozygous	+	Normal	Normal

29	Arab	15	30	F	+	+	+	+	-	Ex:10	c. 1176G>A p. Trp392	Nucleotide substitution	Heterozygous	+	Normal	Normal

30	Asian	37	42	F	+	+	+	-	-	-	c.2118+1G>T	Donor Splice site	Homozygous	NA	NA	NA

31	Caucasian	23	23	M	+	-	-	-	-	-	c.397C>T	NA	Homozygous	+	NA	NA

32	Caucasian	20	20	F	+	-	-	-	-	-	c.397C>T	Mutation in DYSF	Homozygous	+	NA	NA

33	Asian	37	37	M	+	+	+	-	-	-	c.843+1G>C	Donor Splice Site	Homozygous	+	NA	NA

34	Caucasian	13	14	F	+	+	+	-	-	Ex: 13	c.1534C>T p.Arg512Cys	NA	Homozygous	-	Small midbrain and ponds, cerebellar atrophy	Delayed cortical wave defective conduction of large sensory fibers

35 (our case)	Latin American	23	38	F	+	+	+	+	+	In: 12 and 16	c.1729+1G>A c.1353+2T>C	Donor Splice Mutation	Heterozygous	NA	Normal	NA

Abbreviations. F: female, M: male, +: present, -: absent, LoF: loss of function, DYSF: dysferlin, MRI: magnetic resonance image, C: cerebral, S: spinal, NCS: nerve conduction studies, SSEP: somatosensory evoked potentials, NA: not available.

## Data Availability

All data generated or analyzed during the case report are included in the published article.

## Abbreviations

HSP: Hereditary spastic paraplegia
*SPAST*: Spastin gene
*SPG*: Spastic paraplegia gene
*CAPN1*: Calpain 1 gene
MRI: Magnetic resonance imaging.

## Supplementary Materials

Supplementary Materials Video Legends: We can observe interrupted slow horizontal and vertical eye movements with slow horizontal saccades. She had spasticity and hyperreflexia more pronounced in lower extremities. She also showed bilateral Hoffman and Trömner signs, with clonus in lower extremities, and presence of Babinski sign bilaterally. She presented cervical dystonia with laterocollis to the left, and mild bradykinesia was observed during rapid movements. Finger to nose test showed dyssynergia and hypometric movements, rapid alternating movements with dysdiadochokinesia, past pointing, and finger chasing with dyssynergia and dysmetria more pronounced on left upper extremity. Gait was spastic type with scissoring legs.
